# Transient Hypoglossal and Lingual Nerve Injury Following the Use of I-gel Supraglottic Airway: A Case Report

**DOI:** 10.7759/cureus.47509

**Published:** 2023-10-23

**Authors:** Claudia L Sotis, Hosseinali Jafari, Joshua J Solano, Irina Fishman

**Affiliations:** 1 Surgical Services, West Palm Beach Veterans Affairs Medical Center, West Palm Beach, USA; 2 Emergency Medicine, Florida Atlantic University, Boca Raton, USA

**Keywords:** supraglottic airway device, i-gel airway, iatrogenic complication, laryngeal mask airway (lma), lingual nerve palsy, hypoglossal nerve palsy

## Abstract

Injury to the hypoglossal and/or lingual nerve is a rare occurrence with the use of a laryngeal mask airway (LMA) or supraglottic airway (SGA) device. There has been one prior report of a lingual and hypoglossal nerve injury with the i-gel™ SGA. We are describing the second reported hypoglossal and lingual transient nerve injury in a male patient while using an i-gel™ SGA. Although excessive cuff pressure has been cited as a possible cause, the i-gel™ does not have a cuff. This report highlights that hypoglossal nerve injury can still occur, even with the use of a cuffless LMA such as the i-gel™ SGA.

## Introduction

Injury to the lingual or hypoglossal nerves with the use of a laryngeal mask airway (LMA) or subglottic airway (SGA) is quite rare. Using PubMed/Google Scholar, we found only six case reports of lingual nerve neuropraxia and one mention of hypoglossal nerve neuropraxia with the I-gel SGA™. Previous case reports have involved LMAs with inflatable cuffs. The nerve injuries were thought to be associated with over-inflation of the cuff resulting in direct nerve compression. Thus, it was recommended that cuff pressures be monitored continuously during the procedure to avoid such injuries [1].

This case report is unique as we report neuropraxia resulting from a #4 I-gel SGA™ which is devoid of a cuff. The mechanism of injury is unclear. Here, we report both lingual and hypoglossal nerve injury in a male patient who was administered general anesthesia for 100 minutes. This case report raises clinician awareness of possible neuropraxia and briefly discusses available treatments.

## Case presentation

A 54-year-old male patient (weight 97.5 kg, height 173 cm) with a medical history of obstructive sleep apnea using continuous positive airway pressure and obesity with a body mass index of 32.6 kg/m^2^ presented for right wrist surgery for the removal of transcutaneous screws. His preoperative airway examination revealed a Mallampati 3. General anesthesia was induced with 200 mg propofol, 0.1 mg glycopyrrolate (to control excessive secretions), and 50 µg fentanyl. The Student Nurse Anesthetist initially attempted to insert a lubricant-covered #5 i-gel™ but found out quickly that the chosen size was too big. She switched to a lubricant-covered #4 i-gel™ which was easily inserted. There was no evidence of tongue trapping or misalignment. The patient remained in a supine position for the procedure with his head in a neutral position. Assisted ventilation was not required as the patient was breathing spontaneously immediately post-SGA insertion. A leak was not appreciated. Anesthesia was maintained using sevoflurane.

The surgery took longer than anticipated due to the bone having overgrown some of the screws. The patient required 10 mg hydralazine for sustained hypertension which was initially managed with 50 µg fentanyl and increased sevoflurane. The total time from SGA insertion to removal was 100 minutes. On extubation, there was no blood appreciated on the removal of the I-gel.

In the post-anesthesia care unit, the anesthesiologist was called to examine the patient as he was complaining of tongue numbness. On examination, his tongue deviated to the right with extension. The rest of the examination revealed a symmetrical smile, no drooping of the face, no shift of the uvula, and equal lift of the posterior pharynx when eliciting a gag reflex. The tongue deviation to the right and complaint of tongue numbness were the only notable neurologic findings on the examination. There was no oral swelling, hematomas, or erythema on the physical examination. The anesthesia chart was reviewed and there were no appreciable hypotensive episodes so an ischemic stroke was felt to be less likely. A stroke workup was not initiated in light of the very fine neurological deficit appreciated, the lack of hypotension during the case, and the fact that the patient had little cardiac/vascular past medical history. Instead, the patient was discharged home with clear instructions to return to the emergency room if he noticed any further neurological changes or worsening of his symptoms. The next day the anesthesiologist followed up with a phone call during which the patient reported a loss of taste in addition to his original symptoms. A literature search by that time had revealed cases of neuropraxia with the use of other LMAs. An appointment with ENT was arranged for the patient to be evaluated the following week.

A follow-up examination with otolaryngology on postoperative day five showed resolution of tongue deviation but a persistent loss of taste and numbness of the tongue. The diagnosis of lingual nerve injury and resolved hypoglossal nerve injury was established. The patient was prescribed 1,000 mg of B12 BID and 100 mg of B1 QD for one week for the residual tongue numbness. Decadron was not prescribed by the ENT physician as he was seeing him days out from the nerve injury, the tongue was no longer deviated, and there was no appreciable swelling. Another follow-up appointment with ENT was scheduled for two months later. The area of numbness started to improve on postoperative day 25 and was back to baseline on postoperative day 56.

## Discussion

The LMA was invented by Archie Brain, a British Anesthesiologist, and introduced to the market in 1988. The i-gel™ SGA debuted in 2007. It does not have an inflatable cuff and is designed to anatomically fit pharyngeal, laryngeal, and perilaryngeal structures, thus decreasing the risk of nerve injury from compression [2].

A literature search utilizing PubMed/Google Scholar from 2007 to 2023 produced six case reports with injury to the lingual nerve involving the I-gel™ (Table 1). The first reported lingual nerve damage with an I-gel™ was reported in 2011 [3]. They attributed certain design characteristics leading to lingual nerve compression. They surmised that the proximal ridge of the I-gel™ located at the proximal end of the bowl and the wide symmetrical tube stem may be causing excessive pressure at the base and the lateral edge of the tongue.

**Table 1:** Case reports from inception to 2023 involving i-gel LMA with injury to the lingual nerve or hypoglossal nerve. LMA: laryngeal mask airway; TLT: total LMA time; TOT: total operative time; UK: unknown

i-gel™	Size	Duration (minutes)	Gender	Age	Weight (kg)	Position	Symptom	Duration (weeks)	Management
Renes et al. [3], 2011	4	TOT 45	Male	69	78	Supine	Bilateral numbness and loss of taste	8	Conservative
Jenkinson et al. [4], 2014	4	TLT 210	Female	64	UK	Supine	Loss of sensation and taste	>6 90% recovery	UK
Metha et al. [5], 2017	UK	1,440	Female	32	UK	Supine	Dysphagia, numbness, and deviation of the anterior tongue	6	conservative
Rujirojindakul et al. [6], 2012	3	TOT 45	Female	33	53	Supine	Numbness at the tip of the tongue	2	Conservative
Theiler et al. [7] 2012	UK	UK	UK	UK	UK	UK	Bilateral numbness	8	UK
Ueshima et al. [8], 2016	4	UK	Male	53	78	Supine	Bilateral numbness	2	UK

In five of the six cases, full recovery occurred without interventions. In one case, the patient reported 90% recovery to baseline [4]. A literature search from 2007 to 2023 revealed one case report involving the i-gel™ SGA with mention of injury to both the lingual and hypoglossal nerves [5]. The case report by Mehta et al. of injury both to the lingual and hypoglossal nerve is different from this case report because the i-gel™ remained in place for 24 hours before being removed in the previous report [5].

Injury to the hypoglossal nerve is even less common. A review article by Thiruvenkatarajan et al. identified 11 patients with injury to the hypoglossal nerve versus 22 cases of lingual nerve injuries with the use of an LMA from 1988 to April 2014 [9]. Since then, we identified additional case reports [5,10-13]. Of all 16 reported hypoglossal nerve injuries, 14 were with cuffed LMAs, and only two were with cuffless LMAs [5,12].

Although a smaller i-gel™ SGA may cause less compression risk, an LMA or SGA too small has been linked to lingual nerve injury due to malposition. Other proposed causes are the use of nitrous oxide with cuffed LMAs, head/neck/body positional changes, chemical neuritis by the use of chemical lubricant, and local inflammation due to insertion trauma [1]. As the i-gel™ is cuffless, the use of nitrous oxide would not be a contributing factor, nor did we use nitrous oxide in this case. The head, neck, and overall body position remained neutral as the patient remained in the supine position intraoperatively. We used a standard water-soluble lubricant, thus discounting chemical neuritis.

**Figure 1:**
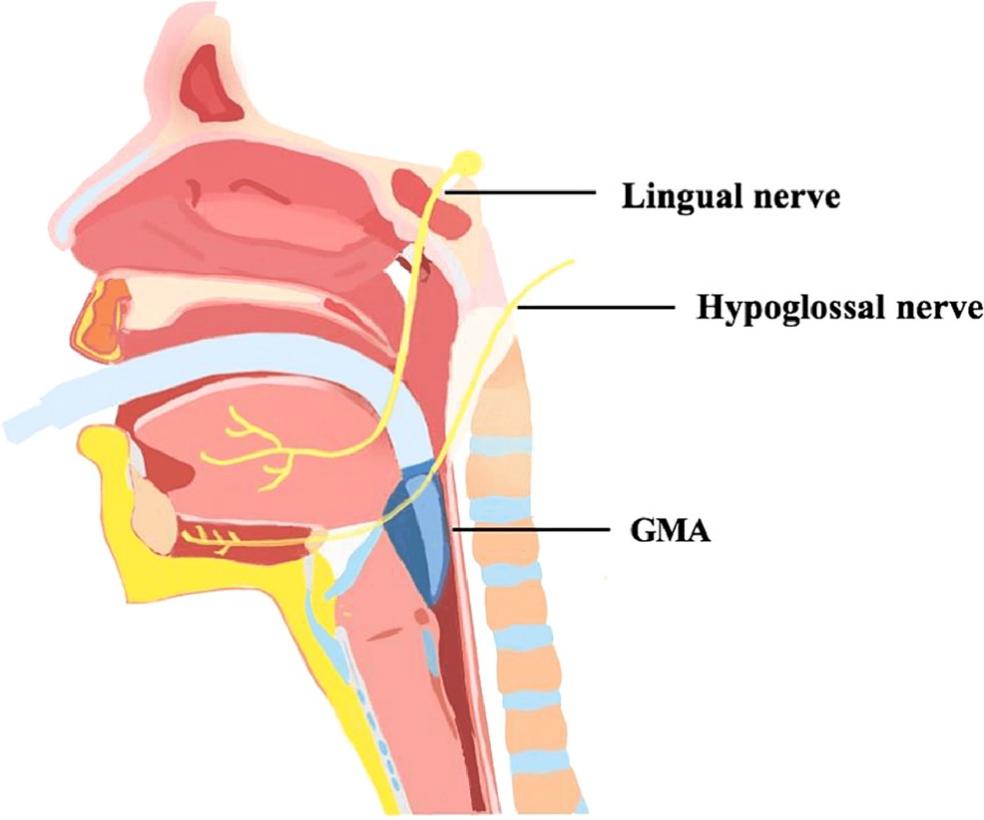
Image of an inserted supraglottic airway demonstrating how it can compress the lingual and hypoglossal nerves. GMA: glottis mask airway Reproduced from Li et al. [12]. This article is available under the Creative Commons CC-BY-NC license and permits non-commercial use, distribution, and reproduction in any medium, provided the original work is properly cited.

The case report by Li et al. is the first case report of lingual and hypoglossal neuropraxia involving a cuffless LMA [12]. Li et al. proposed that positioning of the patient’s head may have played a role in the resulting neuropraxia. Although the patient was in a supine position, the head was in extreme left rotation for surgical convenience [12]. They inferred that the “wide rigid composition at the lower part of the tube, probably resulted in an increase in the pressure on the lingual nerve at the medial aspect of the inner surface of the mandible, which was close to the third molar and hypoglossal nerve at the site of the angle of the mandible” [12]. An image of an I-gel SGA inserted along with the course of the lingual and hypoglossal nerves can be seen in Figure 1 [12]. The visualization helps explain how neuropraxia can occur from nerve compression.

While the mechanism of neuropraxia in our case report is unclear, there are several hypotheses that can provide a feasible explanation. First, our case involved the insertion of a poorly fitting I-gel #5 followed by the insertion of an I-gel #4. Multiple insertion attempts with an SGA make direct trauma to nerves more likely even if no blood was appreciated on the I-gel. Additionally, the lingual nerve is particularly susceptible to injury given its anatomical course. The course of the nerve can be appreciated in Figure 1. This nerve is responsible for sensory innervation of the tongue and taste to the anterior two-thirds of the tongue. The lateral edge of the tongue base and the medial aspect of the mandible next to the third molar is where this nerve is most likely to incur injury. The patient characteristics including obesity, obstructive sleep apnea, and higher Mallampati scores likely created a smaller mouth space which could be associated with nerve injury given the increased likelihood of mechanical compression by the device. Finally, the patient’s weight of 97 kg is close to the recommendations for size #5 or #4 I-gel. Size #5 i-gel was initially chosen based on the manufacturer’s weight recommendation that specifies weights >90 kg. This was downgraded to a size #4 as the #5 appeared too large for the mouth. Size #4 may have been slightly undersized and could have been malpositioned during the case. It is recommended to check LMA positioning periodically throughout the case to prevent any possible malpositioning that could cause neuropraxia.

In summary, our 54-year-old male patient presented with both lingual and hypoglossal nerve injury with the use of an #4 i-gel™ that initially appeared to be optimally positioned for about 100 minutes. A literature review revealed only one prior mention of a hypoglossal injury with this particular SGA [9].

## Conclusions

This case report is unique as it discusses a case of neuropraxia of the lingual and hypoglossal nerves resulting from an I-gel which is a cuffless SGA. Most literature searches highlight such reports in cuffed LMAs. Given the rarity of these injuries with LMAs in general and more specifically with I-gels, we hope to raise clinician awareness of such nerve injuries. We proposed several hypotheses for why this neuropraxia could have occurred in our case. Through appropriate recognition, an expensive neurological workup to rule out CVA can be avoided. Finally, we wanted to emphasize that most cases of such neuropraxia are self-resolving.
